# Deep learning-based relapse prediction of neuromyelitis optica spectrum disorder with anti-aquaporin-4 antibody

**DOI:** 10.3389/fneur.2022.947974

**Published:** 2022-08-05

**Authors:** Liang Wang, Lei Du, Qinying Li, Fang Li, Bei Wang, Yuanqi Zhao, Qiang Meng, Wenyu Li, Juyuan Pan, Junhui Xia, Shitao Wu, Jie Yang, Heng Li, Jianhua Ma, Jingzi ZhangBao, Wenjuan Huang, Xuechun Chang, Hongmei Tan, Jian Yu, Lei Zhou, Chuanzhen Lu, Min Wang, Qiang Dong, Jiahong Lu, Chongbo Zhao, Chao Quan

**Affiliations:** ^1^Department of Neurology, Huashan Rare Disease Center, Huashan Hospital, Shanghai Medical College, Fudan University, Shanghai, China; ^2^National Center for Neurological Disorders (NCND), Shanghai, China; ^3^Department of Neurology, The First Affiliated Hospital of Xinjiang Medical University, Urumqi, Xinjiang Uygur Autonomous Region, China; ^4^Department of Rehabilitation Medicine, Jing'an District Centre Hospital of Shanghai, Fudan University, Shanghai, China; ^5^Department of Rehabilitation Medicine, Huashan Hospital, Shanghai Medical College, Fudan University, Shanghai, China; ^6^Department of Neurology, Jing'an District Centre Hospital of Shanghai, Fudan University, Shanghai, China; ^7^Department of Neurology, The Second Affiliated Hospital of Guangzhou University of Chinese Medicine, Guangzhou, China; ^8^Department of Neurology, The First People's Hospital of Yunnan Province, Kunming, China; ^9^Department of Neurology, Sir Run Run Shaw Hospital, School of Medicine, Zhejiang University, Hangzhou, China; ^10^Department of Neurology, The First Affiliated Hospital of Wenzhou Medical University, Wenzhou, China; ^11^Department of Neurology, The Fifth Affiliated Hospital of Zhengzhou University, Zhengzhou, China; ^12^Department of Neurology, Wuhan No.1 Hospital, Wuhan, China; ^13^Department of Neurology, Central Hospital Affiliated to Shandong First Medical University, Jinan, China; ^14^Department of Ophthalmology and Vision Science, Eye and ENT Hospital, Shanghai Medical College, Fudan University, Shanghai, China

**Keywords:** neuromyelitis optica spectrum disorder, anti-aquaporin-4 antibody, machine learning, deep learning, relapse prediction

## Abstract

**Objective:**

We previously identified the independent predictors of recurrent relapse in neuromyelitis optica spectrum disorder (NMOSD) with anti-aquaporin-4 antibody (AQP4-ab) and designed a nomogram to estimate the 1- and 2-year relapse-free probability, using the Cox proportional hazard (Cox-PH) model, assuming that the risk of relapse had a linear correlation with clinical variables. However, whether the linear assumption fits real disease tragedy is unknown. We aimed to employ deep learning and machine learning to develop a novel prediction model of relapse in patients with NMOSD and compare the performance with the conventional Cox-PH model.

**Methods:**

This retrospective cohort study included patients with NMOSD with AQP4-ab in 10 study centers. In this study, 1,135 treatment episodes from 358 patients in Huashan Hospital were employed as the training set while 213 treatment episodes from 92 patients in nine other research centers as the validation set. We compared five models with added variables of gender, AQP4-ab titer, previous attack under the same therapy, EDSS score at treatment initiation, maintenance therapy, age at treatment initiation, disease duration, the phenotype of the most recent attack, and annualized relapse rate (ARR) of the most recent year by concordance index (C-index): conventional Cox-PH, random survival forest (RSF), LogisticHazard, DeepHit, and DeepSurv.

**Results:**

When including all variables, RSF outperformed the C-index in the training set (0.739), followed by DeepHit (0.737), LogisticHazard (0.722), DeepSurv (0.698), and Cox-PH (0.679) models. As for the validation set, the C-index of LogisticHazard outperformed the other models (0.718), followed by DeepHit (0.704), DeepSurv (0.698), RSF (0.685), and Cox-PH (0.651) models. Maintenance therapy was calculated to be the most important variable for relapse prediction.

**Conclusion:**

This study confirmed the superiority of deep learning to design a prediction model of relapse in patients with AQP4-ab-positive NMOSD, with the LogisticHazard model showing the best predictive power in validation.

## Introduction

Neuromyelitis optica spectrum disorder (NMOSD) is an inflammatory disease of the central nervous system, mainly manifesting as relapsing optic neuritis (ON) and transverse myelitis (TM), resulting in visual and motor disability (1). The majority of patients with NMOSD harbor pathogenic autoantibodies targeting aquaporin 4 (AQP4) water channels in the serum. Disability accumulation in NMOSD is relapse-dependent, thus preventing or delaying relapses is the primary goal in NMOSD management (2). Traditional first-line immunosuppressants used as maintenance therapy in NMOSD include azathioprine (AZA), mycophenolate mofetil (MMF), and rituximab (RTX), while novel monoclonal antibodies such as satralizumab, eculizumab, and inebilizumab have exhibited powerful efficacy in controlling relapses recently (3–7). Recognizing the risk factors of relapse and establishing a suitable prediction model are of utmost importance to inform individualized therapy for NMOSD.

Survival analysis (also called time-to-event analysis) has been widely used to estimate the probability of prognostic outcomes such as death or disease recurrence. The Cox proportional hazard (Cox-PH) model is the most well-known approach for determining the association between a predictive variable of clinical characteristics and the risk of an event such as death (8). Nomogram is a feasible prediction model using risk factors to estimate the probability of a certain event. We previously identified the independent predictors of recurrent relapse in NMOSD, including gender, anti-AQP4 antibody (AQP4-ab) titer, previous attack under the same therapy, EDSS score at treatment initiation, and maintenance therapy, and designed a nomogram to estimate the 1- and 2-year relapse-free probability (9). However, this model was based on the assumption that the risk of a certain event had a linear combination with the variables, which could be too simplistic to fit the actual disease trajectory.

Machine learning is one branch of artificial intelligence and has a wide range of applications, such as predicting carcinoma development and cardiovascular events (10, 11). Compared with the conventional Cox-PH model, machine learning has several advantages, including the ability to continually incorporate new data to optimize algorithms and identify clinically important risks with some marginal variables (12). Deep learning, a novel form of machine learning, employs improved computer power and big data to outperform other machine-learning techniques (13). It has also been used in imaging diagnosis, disease staging, and prognosis and has proved to improve outcome prediction (14–16). The aim of the current investigation was to employ deep learning and machine learning to design a novel prediction model of relapse in patients with NMOSD with AQP4-ab and compare its performance with that of the conventional Cox-PH model.

## Methods

### Study design and participants

This retrospective cohort study included a cohort of patients with NMOSD with AQP4-ab, based on which an outcome prediction model of NMOSD relapse under certain treatment was published by our group (9). We divided NMOSD treatments during follow-up into censored (relapse-free) or failed (disease relapse) treatment episodes. The exclusion criteria were as follows: (1) less than 15 episodes of a certain treatment; (2) lasting for <3 months; (3) without definite start or stop dates; and (4) receiving double/overlapping medications. The effectiveness duration from the last administration time of each medication referred to the study by Stellmann et al. (17).

Among them, 1,135 treatment episodes from 358 patients in Huashan Hospital were employed as the training set while 213 treatment episodes from 92 patients in nine other research centers as the validation set. Each treatment episode was regarded as one independent individual. Demographic and clinical characteristics were collected, including gender, age at treatment initiation, disease duration, AQP4-ab titer, annualized relapse rate (ARR) of the most recent year, Expanded Disability Status Scale (EDSS) score at treatment initiation, the previous attack under same therapy, phenotype of the most recent attack, and maintenance therapy.

All patients from the above research centers received serum AQP4-ab and MOG-ab detection using fixed cell-based indirect immune-fluorescence tests. HEK293 cells transfected with AQP4 M1 isoform or full-length human MOG were employed. AQP4-ab titer ≥1:100 was identified as a high level. MOG-ab was not found in all AQP4 ab-positive patients.

### Statistical analyses

Data analysis and graphing were performed using SPSS version 22.0 (SPSS Inc., Chicago IL, USA), GraphPad Prism 6 (GraphPad Software Inc., La Jolla CA, USA), the rms, survival, and survminer packages of R (version 4.1.3, http://www.r-project.org/). Discrete variables were expressed as count and percentage, while continuous variables were presented as means ± 1 standard deviation or medians with four quantile ranges. Kaplan–Meier survival analysis was used for the relapse curves of the training and validation sets, as well as the training set with various maintenance therapies. Harrell's concordance index (C-index) was deemed as the most suitable and accurate approach for estimating prediction error, which measured the concordance between the predicted and actual probability (18). A Harrell's C-index of 0.5 reveals no predictive discrimination, >0.7 reveals a good model, and > 0.8 reveals a strong model (19). We compared five models with added variables of gender, AQP4-ab titer, previous attack under the same therapy, EDSS score at treatment initiation, maintenance therapy, age at treatment initiation, disease duration, the phenotype of the most recent attack, and ARR of the most recent year by C-index: conventional Cox-PH, random survival forest (RSF), LogisticHazard, DeepHit, and DeepSurv. Statistical significance was set at *p* < 0.05.

### Random survival forest

Building the RSF model and graphing were conducted with RandomForestSRC, ggRandomForests, and ggplot2 packages of R (version 4.1.3, http://www.r-project.org/). It is a nonparametric model that builds hundreds of trees and outputs results in the form of voting (20). This model reduces variance and bias by employing all variables collected and automatically evaluating complex interactions and nonlinear effects (21). The C-index equals the sum of consistent logarithms divided by the total number of data pairs, with a prediction error rate of 1 minus the C-index. The importance of variables was judged by the minimal depth and variable importance (VIMP) method. Higher VIMP or lower minimal depth contributed more to predicting accuracy (22).

### Deep learning

We used three methods of deep learning, which were implemented in Pytorch with the Python package pycox (version 3.7.0, https://www.python.org/). The parameters in the model were manually optimized, with dropouts ranging from 0 to 1. LogisticHazard, also called Nnet-survival, parametrizes the discrete hazards and optimizes the survival likelihood (23). One of the interpolation schemes, called constant hazard interpolation (CHI), was deemed as the C-index. DeepHit is a deep neural network trained by using a loss function, which exploits both relative risks and survival times, whose form of the stochastic process and parameters are dependent on the variables (24). DeepSurv, a multilayer feed-forward neural network, outputs a negative log partial likelihood, which is parameterized by the weights of the network (25). The C-indexes and losses in the training and validation sets were calculated, respectively.

## Results

### Baseline characteristics

The demographic and clinical characteristics of patients with AQP4-ab positive NMOSD in the training and validation sets are demonstrated in Table 1. We included 1,135 counts from Huashan Hospital as the training set and 213 counts from other centers as the validation set. Female patients were the predominant constituent, while approximately one-third of them had the previous attack under the same therapy. ON and TM were the most common recent attacks, with the EDSS score at treatment initiation ranging from 0 to 8.5. More than one-half of the counts had no maintenance therapy or prednisone for <6 months, and more than 10% of the counts had AZA and MMF as their maintenance therapy, respectively.

**Table 1:** The demographic and clinical characteristics of AQP4-ab positive NMOSD patients in the training and validation set.

**Patient characteristics**	**Training set (*n* = 1,135)**	**Validation set (*n* = 213)**
Female gender, n (%)	1,055 (93.0)	198 (93.0)
Age at treatment initiation, years	35.7 (26.2–47.8)	47.5 (34–54.9)
Disease duration, months	18.3 (1.1–49.7)	9.7 (1–29.3)
High AQP4-ab titer (≥1:100), n (%)	643 (56.7)	158 (74.2)
ARR of the most recent year	1 (1–1)	1 (1–2)
EDSS score at treatment initiation	2 (1–3)	2 (1–3)
Previous attack under same therapy, n (%)	419 (36.9)	71 (33.3)
**Most recent attack, n (%)**		
ON	422 (37.2)	40 (18.8)
TM	432 (38.1)	121 (56.8)
Brainstem/cerebral	117 (10.3)	8 (3.8)
Mixed	164 (14.4)	44 (20.7)
**Maintenance therapy, n (%)**		
No or prednisone <6 months	612 (53.9)	110 (51.6)
Prednisone (≥6 months)	19 (1.7)	9 (4.2)
AZA	191 (16.8)	42 (19.7)
MMF	164 (14.4)	27 (12.7)
TAC	46 (4.1)	1 (0.5)
RTX	86 (7.6)	16 (7.5)
CTX	17 (1.5)	8 (3.8)

AQP4-ab, aquaporin-4 antibody; ARR, annualized relapse rate; EDSS, Expanded Disability Status Scale; ON, optic neuritis; TM, transverse myelitis; AZA, azathioprine; MMF, mycophenolate mofetil; TAC, tacrolimus; RTX, rituximab; CTX, cyclophosphamide.

**Figure 1:**
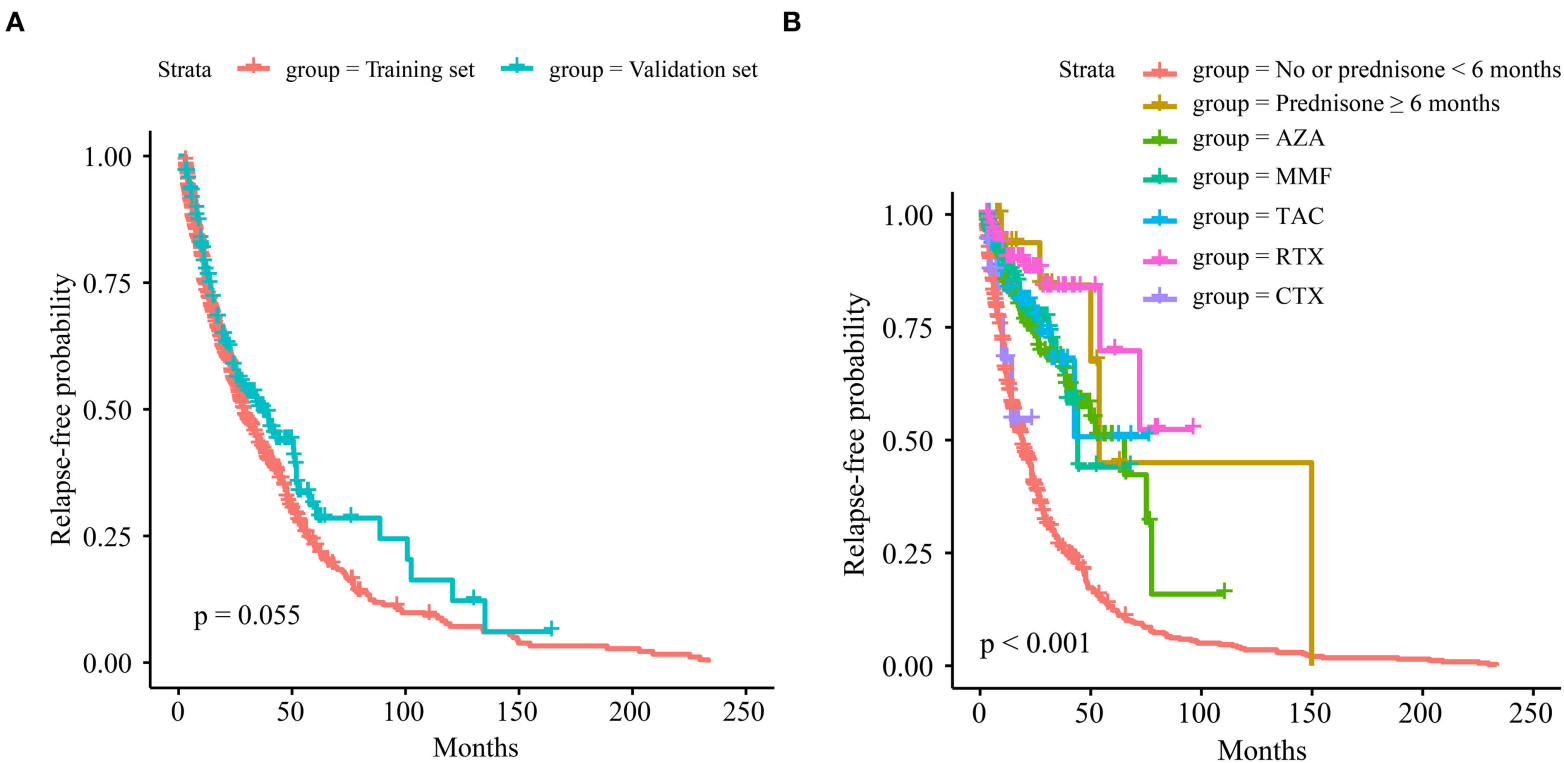
The relapse curves. **(A)** Of the training and validation sets. **(B)** Of each maintenance therapy in the training set.

The relapse curves of the training and validation sets are indicated in Figure 1A, while the difference was not statistically significant (*p* = 0.055). The relapse curves of each maintenance therapy in the training set are shown in Figure 1B, and the difference among them was highly statistically significant (*p* < 0.001). Pairwise comparisons of maintenance therapy using a log-rank test are exhibited in Supplementary Table S1. Among them, compared with no or prednisone <6 months, maintenance therapy of prednisone ≥6 months, AZA, MMF, tacrolimus (TAC), or RTX could efficiently control relapse with high statistical significance (all *p* < 0.001). Compared with CTX, prednisone ≥6 months, AZA, MMF, and RTX could reduce relapse with significance (*p* = 0.017, 0.026, 0.011, and 0.002, respectively). RTX was associated with a statistically significant reduction in relapse when compared with AZA and MMF (*p* = 0.014 and 0.042, respectively).

### Overall comparison

**Figure 2:**
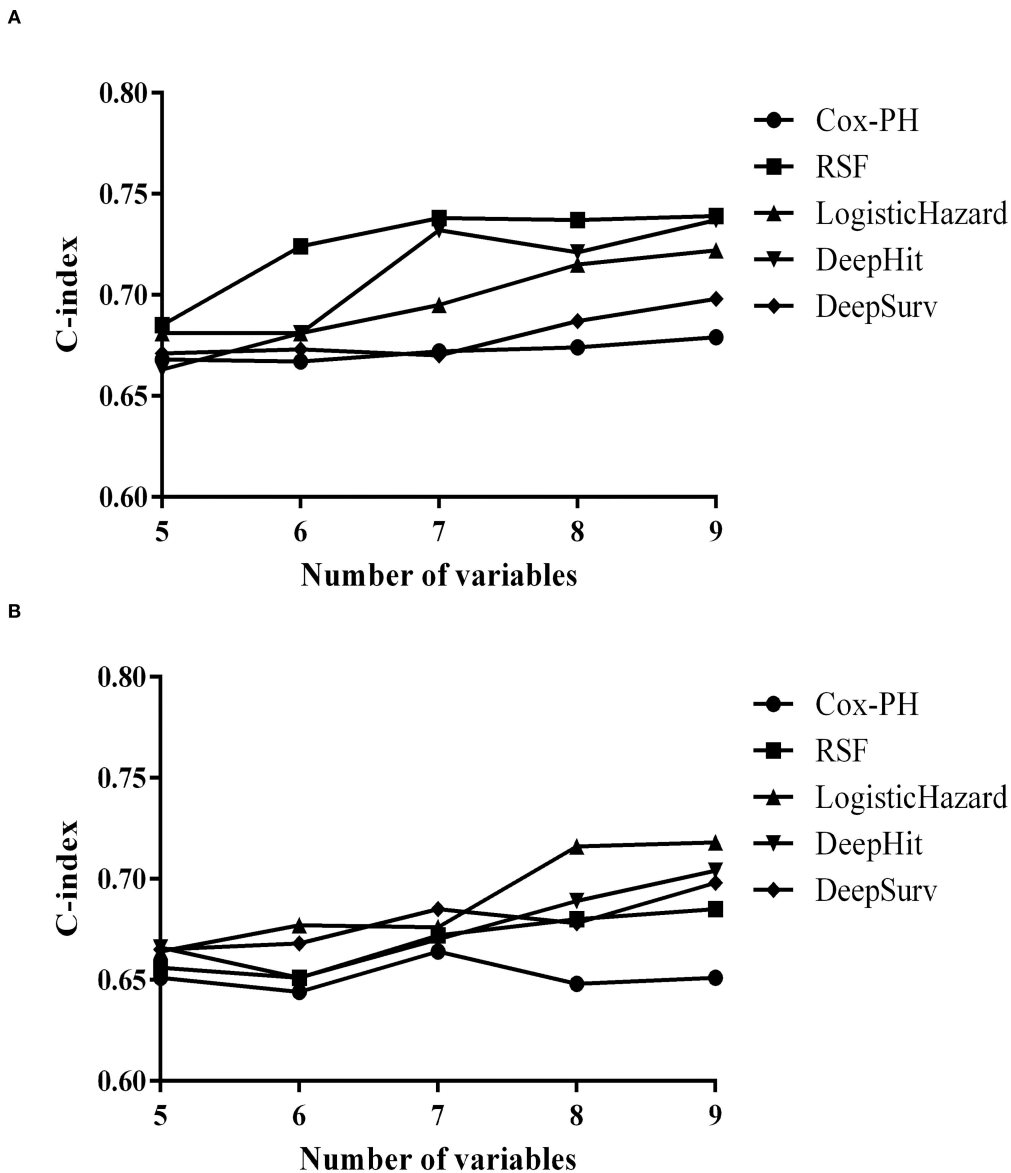
Concordance indexes (C-indexes) in different prediction models with added variables. **(A)** Of the training set. **(B)** Of the validation set.

Prediction models of relapse based on Cox-PH, RSF, LogisticHazard, DeepHit, or DeepSurv patterns were established with the training set and verified in the validation set. The overall comparison of C-indexes with different dropouts using deep learning models in the training set could be seen in Supplementary Figure S1, and the dropout in each model was set at 0.2 according to the optimization results. C-indexes in different prediction models with added variables of the training and validation sets are exhibited in Figures 2A,B.

Initiating with five, the variables were added up to nine incrementally. The first five variables were gender, AQP4-ab, previous attack under the same therapy, EDSS score at treatment initiation, and maintenance therapy, which were statistically significant in our previous study (9). Then, statistically insignificant but clinically important variables were successively included, from age at treatment start, duration of disease, and phenotype of the most recent attack, to ARR of the most recent year.

When including all variables, RSF outperformed the training set (0.739), followed by DeepHit (0.737), LogisticHazard (0.722), DeepSurv (0.698), and Cox-PH (0.679) models. As for the validation set, LogisticHazard outperformed the other models (0.718), followed by DeepHit (0.704), DeepSurv (0.698), RSF (0.685), and Cox-PH (0.651) models.

### Cox-PH model

As the number of variables added up from 5 to 9, the C-index did not increase gradually as some were statistically insignificant variables. Finally, the C-index in the training set rose from 0.668 to 0.679 while in the validation set went up from 0.651 to 0.664 and fell back to 0.651. Independent predictors of relapse with the multivariate Cox-PH model are shown in Table 2, in which AQP4-ab titer, previous attack under the same therapy, EDSS score at treatment initiation, maintenance therapy, and ARR of the most recent year were statistically significant relapse predictors (*p* = 0.003, 0.033, 0.003, < 0.001, and 0.026).

**Table 2:** Independent predictors of relapse with multivariate Cox-PH model.

**Independent predictors**	**Model**	
	**Hazard ratio (95% CI)**	***p*-Value**
Female gender (Reference = male)	1.40 (0.98–1.99)	0.063
AQP4-ab titer (Reference = <1:100)	1.29 (1.09–1.53)	0.003**
Previous attack under same therapy (Reference = no)	1.26 (1.02–1.56)	0.033*
EDSS score at treatment initiation (Reference = <2.5)	0.90 (0.84–0.97)	0.003**
**Maintenance therapy (Reference** **=** **no or prednisone** **<6 months)**		
Prednisone (≥6 months)	0.28 (0.11–0.68)	0.005**
AZA	0.39 (0.29–0.53)	<0.001***
MMF	0.33 (0.23–0.48)	<0.001***
TAC	0.34 (0.19–0.61)	<0.001***
RTX	0.18 (0.10–0.33)	<0.001***
CTX	0.94 (0.38–2.29)	0.89
Age at treatment initiation, years	1.01 (1.00–1.01)	0.11
Disease duration, months	1.00 (1.00–1.00)	0.08
**Phenotype of the most recent attack (Reference** **=** **brainstem/cerebral)**		
ON	0.86 (0.65–1.14)	0.30
TM	0.94 (0.71–1.25)	0.69
Mixed	1.00 (0.72–1.39)	1.00
ARR of the most recent year	1.20 (1.02–1.41)	0.026*

AQP4-ab, aquaporin-4 antibody; EDSS, Expanded Disability Status Scale; AZA, azathioprine; MMF, mycophenolate mofetil; TAC, tacrolimus; RTX, rituximab; CTX, cyclophosphamide; ON, optic neuritis; TM, transverse myelitis; ARR, annualized relapse rate.

* p < 0.05.

** p < 0.01.

*** p < 0.001.

### RSF model

**Figure 3:**
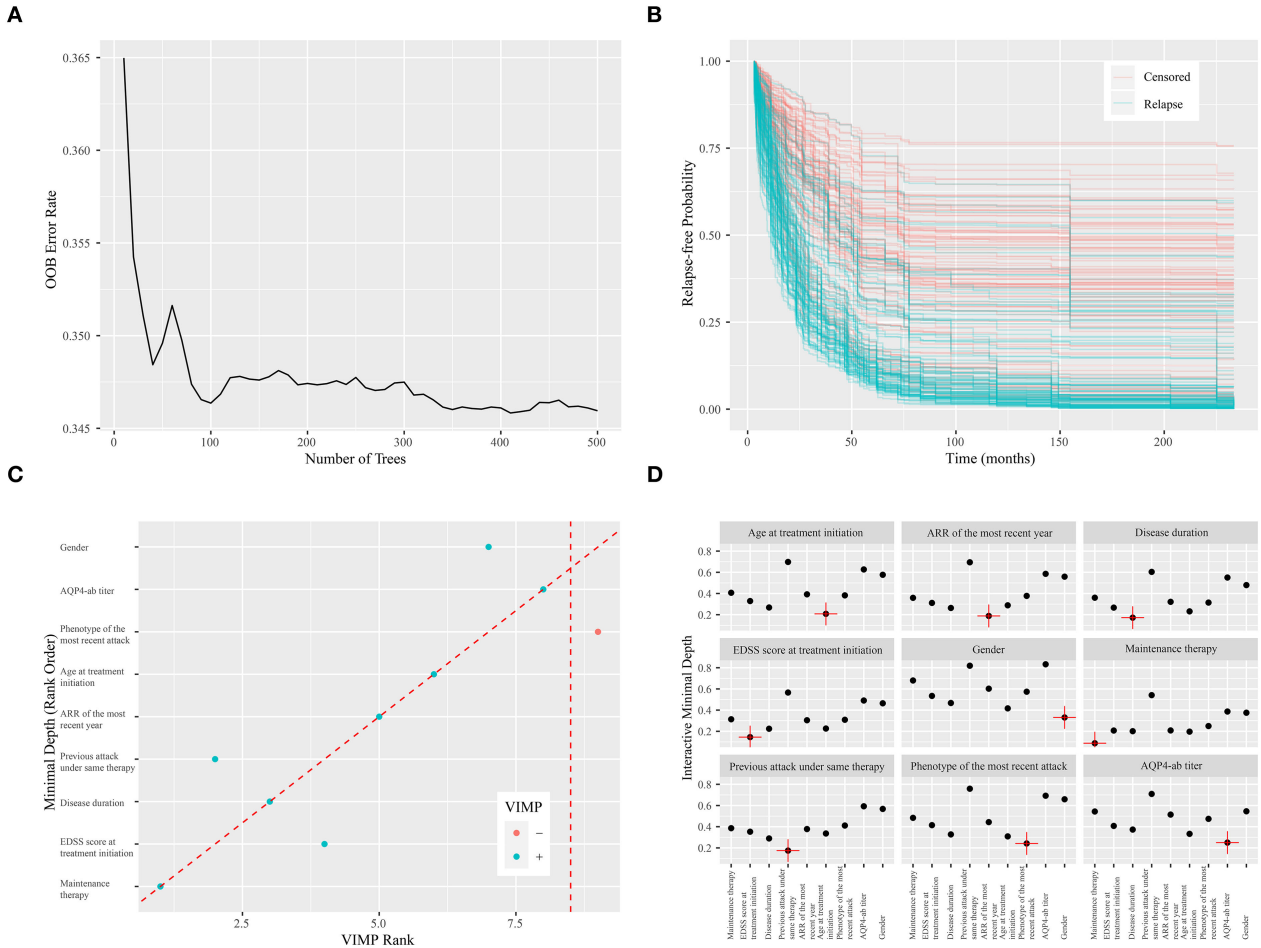
Random survival forest model. **(A)** The error rate of model prediction with different numbers of survival trees. **(B)** Relapse-free estimate for patients in the validation set. The blue line indicates relapse, while the red line indicates censored data. **(C)** A scatter plot of the variables with minimal depth and variable importance (VIMP) method. The blue dot indicates positive VIMP, while the red dot indicates negative VIMP. **(D)** Variable interaction plot for nine variables. Higher values demonstrate lower interactivity, with the target variable labeled in red.

There was a steady increase in the C-index as the number of variables built in the model expanded. Finally, the C-index in the training set increased from 0.685 to 0.739 while in the validation set from 0.656 to 0.685. The error rate of model prediction with different numbers of survival trees is shown in Figure 3A. The model generated a total of 500 binary classification trees. It could be seen that the decreasing trend in the error rate has slowed down significantly. The error rates in the training and validation sets were 0.261 and 0.315, respectively. Relapse-free estimate for patients in the validation set is exhibited in Figure 3B, with the blue line indicating relapse and the red line indicating censored data. Predictors calculated with minimal depth and VIMP from the RSF model are exhibited in Supplementary Table S2. Considering lower minimal depth or higher VIMP contributed more to predicting accuracy, maintenance therapy was regarded as the paramount variable, with a minimal depth of 1.116 and VIMP of 0.213. A scatter plot of the variables with minimal depth and the VIMP method is shown in Figure 3C to provide a comprehensive view of variable rank. A variable interaction plot of minimal depth for nine variables is exhibited in Figure 3D. Considering higher values equal lower interactivity, disease duration seems to have an association with other variables.

### LogisticHazard model

**Figure 4:**
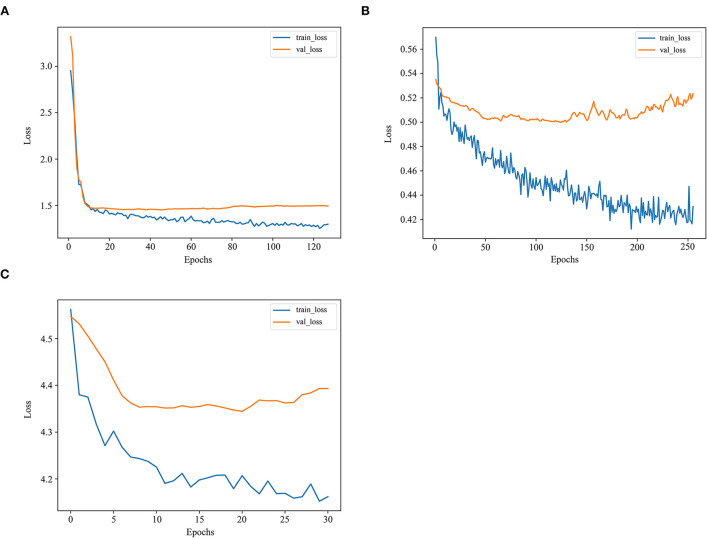
The losses in the training and validation sets with deep learning models. **(A)** The losses in the training and validation sets with the LogisticHazard model. **(B)** The losses in the training and validation sets with the DeepHit model. **(C)** The losses in the training and validation sets with the DeepSurv model.

There was a steady increase in the C-index as the number of variables built in the model expanded. Finally, the C-index in the training set went up from 0.681 to 0.722, while in the validation set from 0.664 to 0.718, the highest among these models. The losses in the training and validation sets with the LogisticHazard model are shown in Figure 4A.

### DeepHit model

As the number of variables added up from 5 to 9, the trend first increased and then fluctuated in the C-index, reaching 0.737 and 0.704 in the training and validation sets, respectively. The losses in the training and validation sets with the DeepHit model are presented in Figure 4B. The loss is the lowest among these deep learning models.

### DeepSurv model

As the number of variables added up from 5 to 9, the C-index first remained steady and then increased, reaching 0.698 and 0.698 in the training and validation sets, respectively. The losses in the training and validation sets with the DeepSurv model are exhibited in Figure 4C. The loss is much higher in this model than in the other two deep learning models.

## Discussion

Previous studies regarding neurology have employed deep learning in stroke diagnosis and outcome prediction, as well as differentiating NMOSD from MS (26, 27). To the best of our knowledge, this was the first study employing artificial intelligence to design the prediction model of relapse in patients with NMOSD with AQP4-ab. It is beneficial for patients to stratify their risk of relapse and avoid ineffective or unnecessary treatment. The deep learning models demonstrated superior predictive power compared with the conventional Cox-PH model, with the LogisticHazard model showing the best predictive power in validation.

The above prediction models included nine variables, namely, gender, AQP4-ab titer, previous attack under the same therapy, EDSS score at treatment initiation, maintenance therapy, age at treatment initiation, disease duration, the phenotype of the most recent attack, and ARR of the most recent year, all of which were common demographical and clinical characteristics. Maintenance therapy was calculated to be the most important variable, which was consistent with the clinical practice (17, 28, 29). Patients with NMOSD underwent more potent immunosuppressive treatment that had less rates of relapse, while those who received disease-modifying treatment had more rates of relapse (3, 28). It emphasized the importance of maintenance therapy as well as the correct differential diagnosis. The previous attack under the same therapy was another important variable, demonstrating that patients with repetitive attacks on certain treatment should convert to a more potent immunosuppressive drug, such as novel monoclonal antibodies such as satralizumab, eculizumab, and inebilizumab. Disease duration, age, clinical manifestation, and ARR before treatment reached statistical significance in other studies and were regarded clinically important to be included in the prediction model (3, 17, 29).

The Cox-PH model is a classical approach to survival analysis and event prediction. However, the model is semiparametric, assuming that the risk of the event has a linear association with the variables. The advantage of the RSF model is that it is not constrained by the assumption of proportional hazard and log-linearity (21). Meanwhile, it could prevent the overfitting problem of its algorithm through two random sampling processes (30). The deep learning model could learn and infer high-order nonlinear combinations between patient outcomes and variables in a fully data-driven manner. Recently, it was demonstrated that deep neural network outperforms standard survival analysis, one of whose advantages is the ability to discern relationships without prior selection of features (25). In this study, the RSF model fits best in the training set but not in the validation set, which may demonstrate some overfitting in this model. The LogisticHazard model performs best in the validation set with added variables, indicating its superior performance and improvements compared with the other models. The loss may partially explain the relative inferiority of the C-index in the DeepSurv model.

The primary limitation was the retrospective nature of this study, with some recall bias. First, the dose of maintenance therapies was varied and not standardized to the same level. Second, AQP4-ab titers were not examined at fixed timing after NMOSD onset. The black-box essence of the deep learning model limited its clinical utility. Future prospective and large-sample studies with advanced technology will further prompt to evolution and visualization of the model.

To conclude, this study confirmed the superiority of deep learning to design a prediction model of relapse in patients with AQP4-ab-positive NMOSD, with the LogisticHazard model showing the best predictive power in validation. Based on the above-optimized model, a personalized treatment recommender system could be developed to minimize the probability of relapse in the future.

## Data availability statement

The raw data supporting the conclusions of this article will be made available by the authors, without undue reservation.

## Ethics statement

The studies involving human participants were reviewed and approved by Medical Ethics Committee of Huashan Hospital. The patients/participants provided their written informed consent to participate in this study.

## Author contributions

LW designed and conceptualized the study, interpreted and analyzed the data, and drafted and revised the manuscript for intellectual content. LD, QL, FL, BW, YZ, QM, WL, JP, JX, SW, JY, HL, and JM provided the data of the validation cohort. JZ, WH, XC, HT, JY, and LZ provided the data of the primary cohort and revised the manuscript for intellectual content. CL, MW, QD, JL, and CZ revised the manuscript for intellectual content. CQ designed and conceptualized the study, interpreted and analyzed the data, and revised the manuscript for intellectual content. LW and CQ conducted the statistical analyses of this manuscript. The corresponding author had full access to all the data in this study and had final responsibility for the decision to submit it for publication. All authors contributed to the article and approved the submitted version.

## Funding

This study was supported by the National Natural Science Foundation of China (Grant No. 82171341), the Shanghai Municipal Science and Technology Major Project (No. 2018SHZDZX01) and ZHANGJIANG LAB, and the National Key Research and Development Program of China (2016YFC0901504).

## Conflict of interest

The authors declare that the research was conducted in the absence of any commercial or financial relationships that could be construed as a potential conflict of interest.

## Publisher's note

All claims expressed in this article are solely those of the authors and do not necessarily represent those of their affiliated organizations, or those of the publisher, the editors and the reviewers. Any product that may be evaluated in this article, or claim that may be made by its manufacturer, is not guaranteed or endorsed by the publisher.
