# Supplementary material for: Deep learning-based relapse prediction of neuromyelitis optica spectrum disorder with anti-aquaporin-4 antibody
Source: Front Neurol. 2022 Aug 5;13:947974. doi: 10.3389/fneur.2022.947974 (PMC9389264; doi:10.3389/fneur.2022.947974)
Supplement: Supplementary file 1 [file Data_Sheet_1.pdf]

Supplemental data to the article: 'Deep Learning-Based Relapse Prediction of Neuromyelitis Optica Spectrum Disorder with Anti-Aquaporin-4 Antibody'

Table S1. Pairwise comparisons of maintenance therapy using log-rank test

Maintenance therapy	No or prednisone < 6 months	Prednisone ≥ 6 months	AZA	MMF	TAC	RTX	CTX
Prednisone > 6 months	< 0.001***	—	—	—	—	—	—
AZA	< 0.001***	0.225	—	—	—	—	—
MMF	< 0.001***	0.223	0.695	—	—	—	—
TAC	< 0.001***	0.369	0.735	0.946	—	—	—
RTX	< 0.001***	0.787	0.014*	0.042*	0.12	—	—
CTX	0.869	0.017*	0.026*	0.011*	0.07	0.002**	—

AZA, azathioprine; MMF, mycophenolate mofetil; TAC, tacrolimus; RTX, rituximab; CTX, cyclophosphamide. *p < 0.05, **p < 0.01, ***p < 0.001.

Table S2. Predictors calculated with minimal depth and VIMP from random survival forest model

Predictors	Minimal depth	VIMP
Maintenance therapy	1.116	0.213
EDSS score at treatment initiation	1.9	0.028
Disease duration, months	2.24	0.033
Previous attack under same therapy	2.328	0.038
ARR of the most recent year	2.418	0.017
Age at treatment initiation, years	2.684	0.011
Phenotype of the most recent attack	3.138	-0.004
AQP4-ab titer	3.266	0.003
Gender	4.276	0.01

VIMP, variable importance; EDSS, Expanded Disability Status Scale; ARR, annualized relapse rate; AQP4-ab, aquaporin-4 antibody.

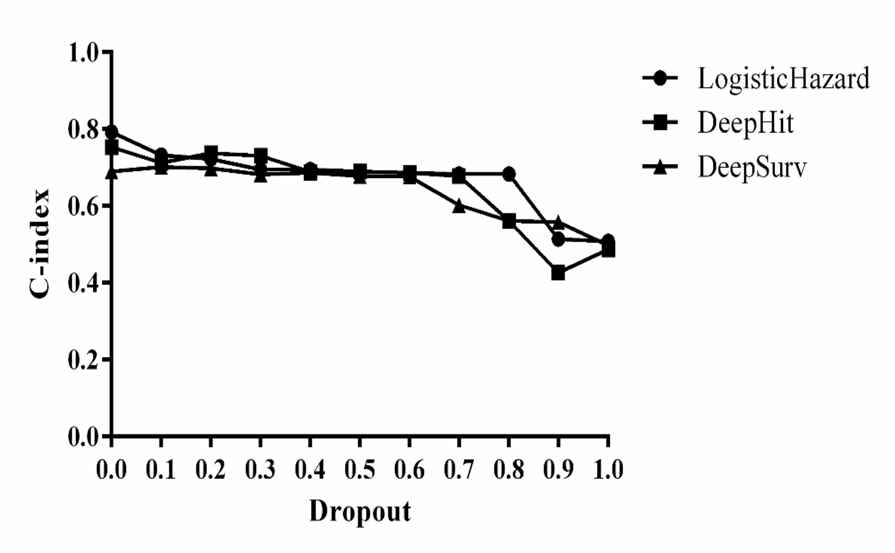

Fig S1. The overall comparison of C-indexes with different dropouts using deep learning models in the training set.
